# Interfacial Assemble of Prussian Blue Analog to Access Hierarchical FeNi (oxy)-Hydroxide Nanosheets for Electrocatalytic Water Splitting

**DOI:** 10.3389/fchem.2022.895168

**Published:** 2022-04-27

**Authors:** Jinquan Hong, Jiangquan Lv, Jialing Chen, Lanxin Cai, Mengna Wei, Guoseng Cai, Xin Huang, Xiaoyan Li, Shaowu Du

**Affiliations:** ^1^ Minjiang Collaborative Center for Theoretical Physics, College of Physics and Electronic Information Engineering, Minjiang University, Fuzhou, China; ^2^ Fujian Key Laboratory of Functional Marine Sensing Materials, College of Physics and Electronic Information Engineering, Minjiang University, Fuzhou, China; ^3^ College of Electronics and Information Science, Fujian Jiangxia University, Fuzhou, China

**Keywords:** FeNiOOH nanosheet, Prussian blue analog, oxygen evolution, hydrogen evolution, water splitting

## Abstract

Developing facile methods for the synthesis of active and stable electrocatalysts is vitally important to realize overall water splitting. Here, we demonstrate a practical method to obtain FeNiOOH nanosheets on nickel foam (NF) as bifunctional electrocatalyst by growing a FeCo Prussian blue analog with further *in situ* oxidation under ambient conditions. The binder-free, self-standing FeNiOOH/NF electrode with hierarchical nanostructures requires low overpotentials of 260 mV and 240 mV at a current density of 50 mA cm^−2^ for oxygen evolution reaction and hydrogen evolution reaction, respectively, in 1.0 M KOH solution. Therefore, an alkaline water electrolyzer constructed by bifunctional FeNiOOH/NF electrode as both anode and cathode delivers 50 mA cm^−2^ under a cell voltage of 1.74 V with remarkable stability, which outperforms the IrO_2_-Pt/C-based electrolyzer. The excellent performance could be ascribed to the superior FeNiOOH intrinsic activity and the hierarchical structure. This work provides a cost-efficient surface engineering method to obtain binder-free, self-standing bifunctional electrocatalyst on commercial NF, which could be further extended to other energy and environment applications.

## Introduction

Water electrolysis provides a feasible and sustainable solution to replace fossil fuels by carbon-neutral hydrogen (H_2_) for future renewable-energy scenario (Kibsgaard and Chorkendorff, 2019; Cao et al., 2020; Hongming Sun et al., 2020; Jiang et al., 2020; Shen Zhang et al., 2020; Su et al., 2021). The electrolyzer requires highly efficient and stable electrocatalysts to drive two half reactions: hydrogen evolution reaction (HER) and oxygen evolution reaction (OER). Particularly, the multistep proton and electron transfer processes make OER more sluggish, which greatly restricts the water electrolysis efficiency (Yan et al., 2018; Hu et al., 2019). Although the benchmark electrocatalysts for HER and OER are Pt-based and Ir, Ru-based materials, respectively, their high cost and scarcity as well as the instability greatly hamper the practical applications (Yang et al., 2021; Guan et al., 2022). Moreover, the single-function noble metal electrocatalysts require different preparation and optimization procedures for integrated electrolyzer (Wu et al., 2016). Therefore, the development of highly active and durable, low-cost and readily useable bifunctional electrocatalysts is highly desired.

Over the past few years, great progress has been achieved in developing transition-metal-based HER and OER bifunctional electrocatalysts, including transition-metal alloys (Xinsha Zhang et al., 2020), oxides (Huang et al., 2019), (oxy)hydroxides (Lei et al., 2020; Bin Liu et al., 2018; Govind Rajan et al., 2020; Govind Rajan and Carter, 2020; Hu et al., 2022), sulfide/selenides (Chen et al., 2020), and nitride/phosphides (Su et al., 2017). Among them, the FeNi-based (oxy)hydroxides (FeNiOOH) have been considered as a promising candidate due to their excellent OER activity and durability (Gong et al., 2013; Lu and Zhao, 2015). Moreover, detailed mechanism studies have demonstrated that FeNiOOH was the real catalytically active species in many types of OER electrocatalysts generated from irreversible surface reconstruction (Jin, 2017; Yu et al., 2018). However, FeNiOOH showed a relatively low activity for HER, limiting their practical application as bifunctional catalyst for water splitting (Hu et al., 2017; Yipu Liu et al., 2018). Additionally, the inherently poor conductivity of FeNiOOH as well as the using of nonconductive Nafion binders results in poor performance of the entire electrode (Zou et al., 2015; Park et al., 2019). On the other hand, the added conductive carbon black inclines to degrade during long-term cycles, which impairs the stability (Katsounaros et al., 2014; Zuo et al., 2019). Therefore, constructing free-standing integrated electrodes by *in situ* growing FeNiOOH on conductive substrates may offer a feasible solution to achieve high efficiency and excellent stability for overall water splitting (Zhao Sun et al., 2020).

Until now, there are only a few reports on direct preparation of FeNiOOH on conductive substrates as bifunctional electrocatalysts for water splitting. For example, Yangdo Kim et al. presented a galvanic corrosion method and a hydrothermal method to prepare sulfur-incorporated FeNiOOH on commercial nickel foam (NF), which required high temperature (Kim et al., 2022). Chemical transformation is a simpler method to obtain various structures as desired under ambient conditions. Zhiqiang Wang et al. reported a facile method to synthesis FeNiOOH nanosheets on FeNi foam (FNF) by *in-situ* chemical oxidation for OER, which took a long-time reparation process (Wang et al., 2020). Especially, Prussian blue analogs (PBAs), which can be synthesized under room temperature in aqueous solution, have been regarded as promising precursors for the synthesis of various types of materials, e.g., transition metal alloys, oxides, sulfides, (oxy)hydroxides, etc. (Yang et al., 2015; Wang et al., 2018a; Xuan et al., 2019; Lv et al., 2021). Shuo Zhang and coauthors reported NiFe PBAs could be transformed into NiOOH under applied potential (Su et al., 2018). However, it is difficult to scale up and remove the additional binders and carbon supports through electrochemical oxidation method. Therefore, by using specific oxidants, which holds the equal oxidizing capacity with applied potentials in electrosynthesis, FeNiOOH could be synthesized by the oxidation of Fe-doped FeNi-PB on NF under ambient conditions. However, none of the research work has achieved this process.

In this study, we first presented a sustainable chemical oxidation route to obtain FeNiOOH nanosheets on NF as bifunctional electrocatalyst for water splitting. The Na_2_NiFe(CN)_6_ (FeNi-PB) nanoparticles could *in situ* grow on NF and exchange with Fe^3+^ ion. Further oxidation with NaClO solution induced the structure reconstruction into FeNiOOH nanosheets on NF. The as-prepared self-standing FeNiOOH/NF electrode with hierarchical nanostructures exhibited excellent OER and HER performance, with low overpotentials of 260 mV and 240 mV at a current density of 50 mA cm^−2^ for OER and HER, respectively, in 1.0 M KOH solution. Therefore, the two-electrode configuration alkaline water electrolyzer with bifunctional FeNiOOH/NF electrode as both anode and cathode delivers 50 mA cm^−2^ under a cell voltage of 1.74 V with remarkable stability, which outperforms the IrO_2_-Pt/C-based electrolyzer. This work not only provides a cost-efficient surface engineering method to obtain binder-free, self-made bifunctional electrocatalyst on commercial NF, but also provides a novel strategy for their potentially scalable preparation at low cost.

## Experimental Section

### Materials

All reagents were purchased from Sinopharm Chemical Reagent Co. Ltd. and used without further purification. Milli-Q water (>18 MΩ cm) was used for preparation and washing.

### Preoxidation of Nickel Foam

Typically, pieces of NF (1cm×3 cm) were cleaned in acetone, 0.5 M HCl, and deionized (DI) water successively through sonication treatment for 10 min each time. The clean NFs were then transferred into round-bottom flask with 3 M HCl aqueous solution, and soaked for 10 min at 90°C. They further washed with DI water several times to remove the residual solution. Finally, the NFs were placed in ambient air 12 h for oxidation.

### Preparation of Fe-Doped FeNi-PB on Nickel Foam

Typically, two pieces of NF were vertically immersed into 20 ml of 0.02 M sodium hexacyanoferrate (Yu et al.) solution with 0.5 M sodium citrate. Then the solution was stilled for 18 h at room temperature for *in situ* growth. The pieces were washed with distilled water and dried at room temperature and named as FeNi-PB-NF. FeNi-PB-NF was vertically put into 15 ml solution containing 0.1 mmol FeCl_3_·6H_2_O with stirring at 600 rpm. The exchange reaction was conducted for 4 h with the color changed from yellow to brown yellow. The pieces were washed with distilled water and dried at room temperature for further use and named as Fe-doped PB-NF.

### Preparation of FeNiOOH Nanosheets on Nickel Foam

In a typical synthesis of FeNiOOH nanosheets on NF (FeNiOOH-NF), Fe-doped PB-NF was immersed into 0.1 M NaClO solution and reacted for 30 min with stirring at 300 rpm. After finishing the reaction, the color of pieces changed from brown yellow to black. The pieces were washed with distilled water and dried at room temperature for further use and named as FeNiOOH-NF. NiOOH on NF was synthesized using FeNi-PB-NF for further oxidation with the same procedure.

### Material Characterization

The morphologies of the as-prepared samples were observed by scanning electron microscopy (SEM, S4800/Cryo). The composition of the samples and elemental mapping were analyzed by energy-dispersive X-ray spectroscope (EDX) attached to the SEM instrument. Transmission electron microscope (TEM) was obtained by a JEM-2100F instrumentation acceleration voltage of 200 kV. The elements on the surface of the as-prepared samples were identified by X-ray photoelectron spectroscopy (XPS, Perkin Elmer PHI 5000 C ESCA) with a monochromic Al/Ka X-ray source. X-ray diffraction (XRD) was performed on a MiniFlex diffractometer with Cu Kα radiation (λ = 1.54 Å).

### Electrochemical Measurements

Electrochemical measurements were performed in a three-electrode electrochemical setup using a computer-controlled electrochemistry workstation (CHI 660E, CH Instrument Inc.). The as-prepared self-standing NF electrodes were directly used as working electrodes. Pt/C and IrO_2_ were loaded on NF with a mass loading of 1 mg/cm^2^ as working electrodes. A platinum wire electrode and an Ag/AgCl saturated KCl electrode served as the counter and reference electrode, respectively. Linear sweep voltammetry (LSV) tests were conducted in 1 M KOH solution at a scan rate of 10 mV s^−1^. Potentials reported in this study were all quoted against the reversible hydrogen electrode (RHE) using equation E vs. RHE = E (Ag/AgCl) + 0.197 + 0.059 × pH. All polarization curves were corrected with 90% iR-compensation.

### Water Splitting Devices and Measurements

Overall water splitting measurements were performed in a two-electrode system consisting of FeNiOOH-NF as both cathode and anode. Pt/C and IrO_2_ were loaded on NF with a mass loading of 1 mg/cm^2^ as cathode and anode, respectively, for comparisons. LSV test was performed in 1 M KOH solution at a scan rate of 5 mV s^−1^. The long-term stability of electrocatalyst was measured by chronopotentiometry test at 50 mA cm^−2^.

## Results and Discussion

**FIGURE 1:**
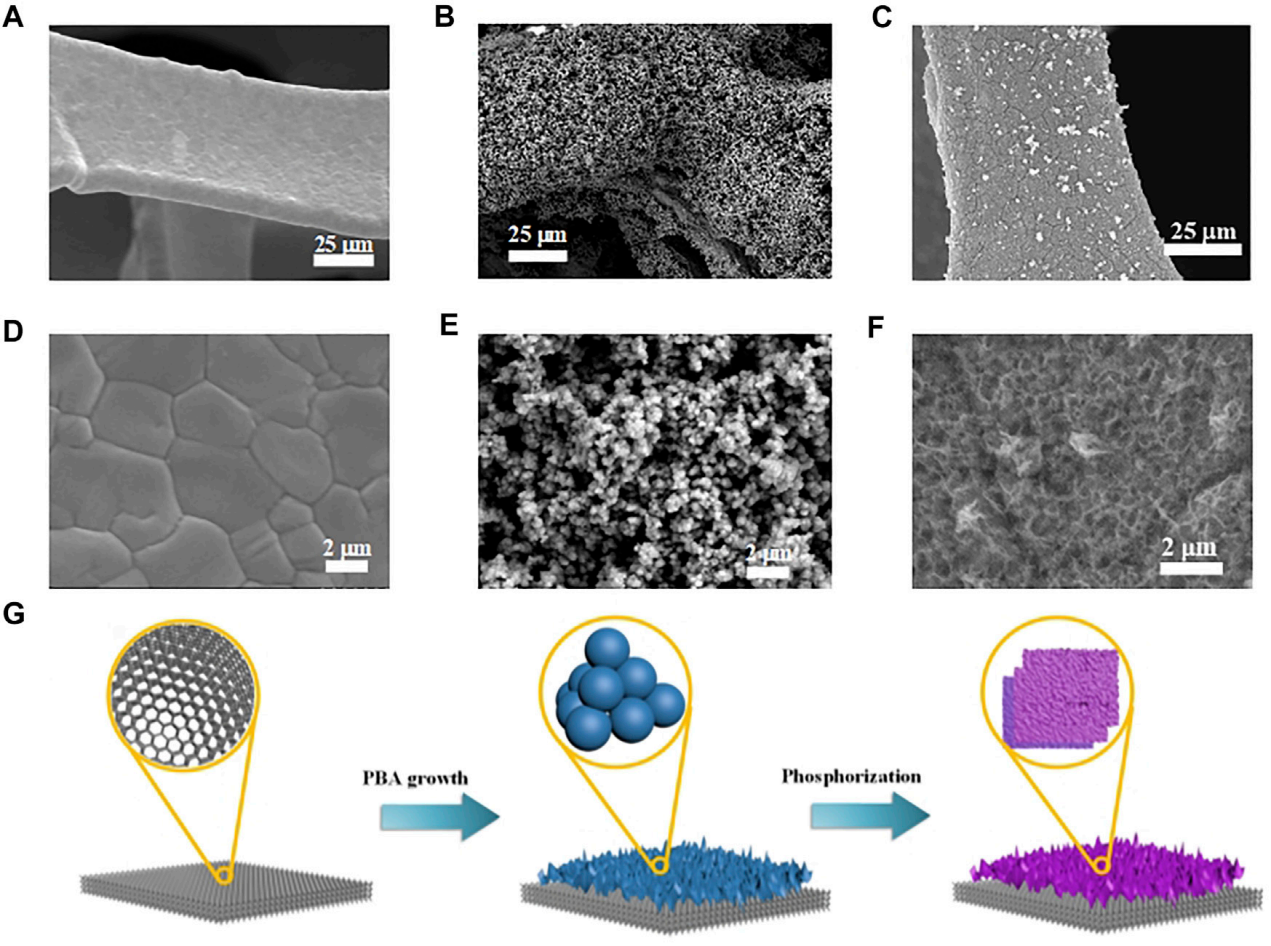
**(A)** and **(D)** SEM images of cleaned NF in different magnifications. **(B)** and **(E)** SEM images of Fe-doped FeNi-PB on NF in different magnifications. **(C)** and **(F)** SEM images of FeNiOOH on NF in different magnifications. **(G)** Schematic for the synthesis processes of FeNiOOH on NF.

Due to the high electrical conductivity and 3D macroporous feature, commercial NF was chosen as supporting substrate for further synthesis. As shown in Figures 1A,D, the cleaned NF showed continuous crystalline grain in the ligaments, resulting in smooth surface without pore and wrinkle. The Fe-doped FeNi PB nanoparticles were directly grown on the surface of NF through a gentle coprecipitation and ion exchange method (Figure 1B). Fe-doped FeNi PB nanoparticles showed uniform sizes about 300 nm, which homogeneously covered the surface of NF and formed 3D macroporous structure (Figure 1E). The FeNiOOH nanosheets could be obtained by a simple and fast (30 min) chemical oxidation route with NaClO oxidant. The FeNiOOH nanosheets were interweaved each other to form a 3D network structure (Figures 1C,F), which enhanced the mechanical stability of electrode for further applications. During the whole synthesis procedures, the visible surface color of NF was gradually changed from metallic luster of NF to brown yellow of Fe-doped FeNi-PB and finally to black of FeNiOOH nanosheets (Supplementary Figure S1). The schematic for the overall evolution processes of FeNiOOH nanosheets on NF based on the above morphology characterizations is presented in Figure 1G. Notably, to synthesize FeNiOOH nanosheets, researchers usually doped Fe in NiOOH nanosheets. Considering the more uniform contribution by ion exchange, we introduced Fe before oxidation process in our synthesis. The possible processes for this transformation reaction were proposed as follows: the subsequent oxidation using strong oxidant NaClO would break the coordination of Ni^2+^ and [Fe(CN)_6_]^4−^and led to the formation of NiOOH nanosheets with the corporation of Fe^3+^ to form FeNiOOH nanosheets.

**FIGURE 2:**
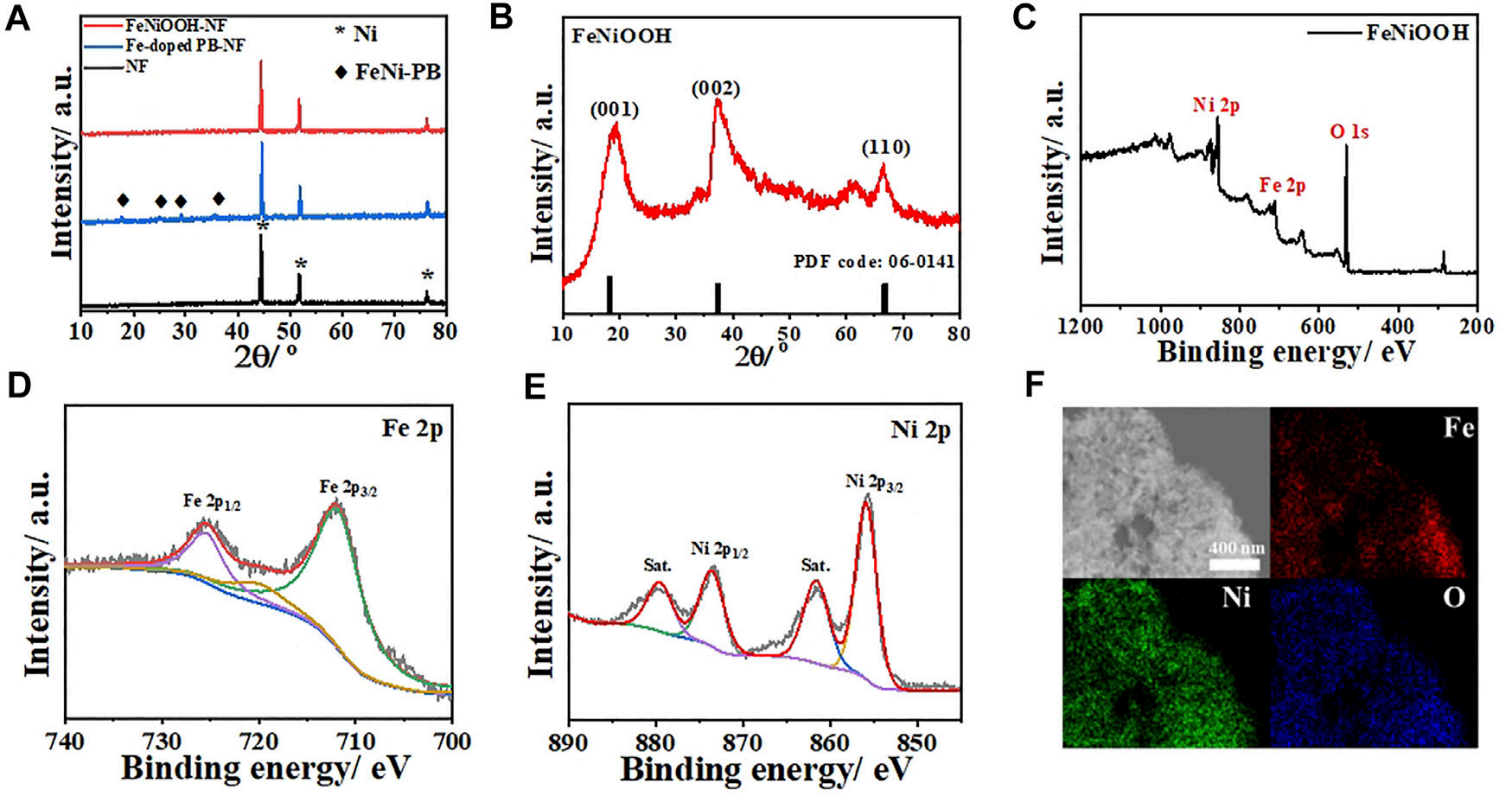
**(A)** XRD patterns of NF, Fe-doped FeNi-PB on NF and FeNiOOH on NF. **(B)** XRD pattern of FeNiOOH scraped from FeNiOOH-NF. **(C)** XPS survey of FeNiOOH scraped from FeNiOOH-NF. **(D)** High-resolution Fe 2p spectra. **(E)** High-resolution Ni 2p spectra. **(F)** Element mapping images of FeNiOOH nanosheets with Fe, Ni and O.

XRD characterizations were conducted to better confirm the structure changes during these synthesis processes. As shown in Figure 2A, the cleaned NF showed typical peaks at 44.6°, 51.9°, and 76.6° (JCPDS: 70–0989). After the growth of Fe-doped FeNi-PB, obvious characteristic peaks at 17.8°, 25.1°, 29.1°, and 35.8° were observed from the XRD pattern, which matched well with the standard diffraction peaks of FeNi-PB (JCPDS: 75–0037). The peaks for NF were kept, which demonstrated the successful synthesis of FeNi-PB on NF. After the oxidation of NaClO, all the peaks of FeNi-PB disappeared. Meanwhile, there were no other characteristic peaks such as nickel oxide except for NF, which indicated that the oxidation reaction happened on Fe-doped FeNi-PB rather than NF. We speculated that diffraction intensity of FeNiOOH nanosheets was strongly weaker than that of NF, which resulted in the disappearance of diffraction peaks of FeNiOOH nanosheets. Therefore, to further confirm the formation of FeNiOOH, we carefully scraped the samples from NF and collected the powders for XRD test. As shown in Figure 2B, the characteristic peaks at 18.4°, 37.3°, and 66.8° could be assigned to (001), (002), and (110) of β-NiOOH structure (JCPDS: 06–0141). Meanwhile, the intensities of (001) and (002) were much higher than that of (110), which indicated the formation of nanosheets structure. Above characterizations demonstrated the successful *in situ* synthesis of the FeNi-PB on NF and the transformation of PBAs into β-NiOOH structure.

**FIGURE 3:**
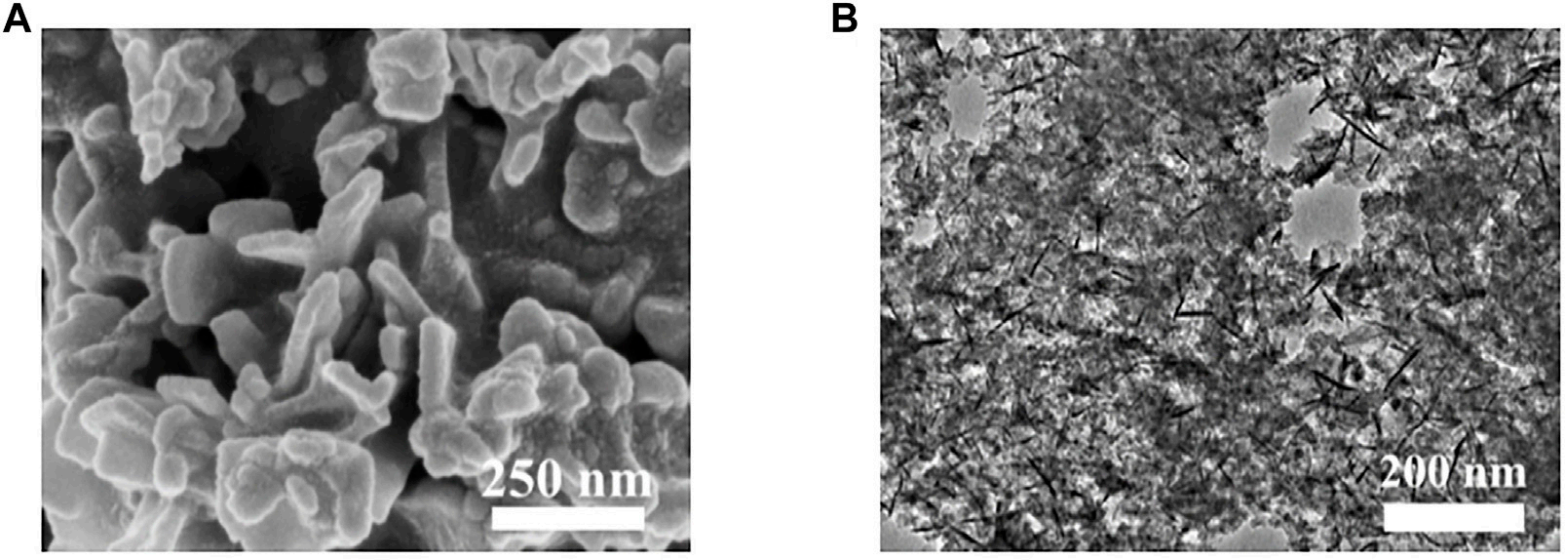
**(A)** SEM image and **(B)** TEM image of FeNiOOH nanosheets scraped from FeNiOOH-NF.

To further confirm the effective introduction of Fe into β-NiOOH structure and investigate the valence state of Fe and Ni in FeNiOOH nanosheets, X-ray photoelectron spectroscopy (XPS) tests were conducted. In the XPS survey spectrum of FeNiOOH-NF (Figure 2C), typical peaks of Fe 2p, Ni 2p, and O 1s could be found and the peak at about 284.8 eV could be assigned to C 1s peak as internal standard for binding energy calibration. The high-resolution XPS profiles of Fe and Ni were shown in Figures 2D,E. In the Fe 2p XPS spectra, the Fe 2p_3/2_ peak at around 712.1 eV together with the Fe 2p_1/2_ peak at 725.6 eV indicated Fe^3+^ oxidation state (Li et al., 2019). The high-resolution XPS spectrum of Ni 2p showed two spin–orbit peaks at 856.0 eV (Ni 2p_3/2_) and 873.8 eV (Ni 2p_1/2_), along with two satellite peaks, which correspond to the Ni^3+^ oxidation state (Wang et al., 2016). We also analyzed the O 1s XPS spectrum of FeNiOOH/NF. As shown in Supplementary Figure S2, the three fitted peaks at 529.5, 531 and 532.5 eV were attributable to the lattice oxygen (Fe/Ni–O), terminal hydroxyl (Fe/Ni–OH), and absorbed water from FeNiOOH nanosheets, respectively (Wang et al., 2018b). These results demonstrated that the FeNi-PB could be completely transformed into NiOOH nanosheets and Fe^3+^ was successful introduced into NiOOH nanosheets. As shown in Figure 2F, energy dispersive X-ray spectroscopy (EDS) elemental mapping images of FeNiOOH nanosheets provided further evidence for the interweaved 3D porous nanostructure and revealed the existence and uniform distribution of Fe, Ni, and O elements in FeNiOOH nanosheets. To more clearly show the detailed information of the nanosheets morphology of FeNiOOH, SEM and transmission electron microscope (TEM) characterizations were conducted by the product peeled off from the FeNiOOH/NF by ultrasonication. Under higher magnifications (Figure 3A), the nanosheets showed about 250 nm in size and 50 nm in thickness. Transmission electron microscope (TEM) was employed to get more insight into the microstructure of FeNiOOH nanosheets (Figure 3B). The continuously interconnected nanosheets with porous architecture can be further confirmed, which is beneficial for mass transfer and gas release. Therefore, above characterizations showed that the Fe^3+^ was successfully introduced into NiOOH to form interconnected FeNiOOH nanosheets with porous architecture.

**FIGURE 4:**
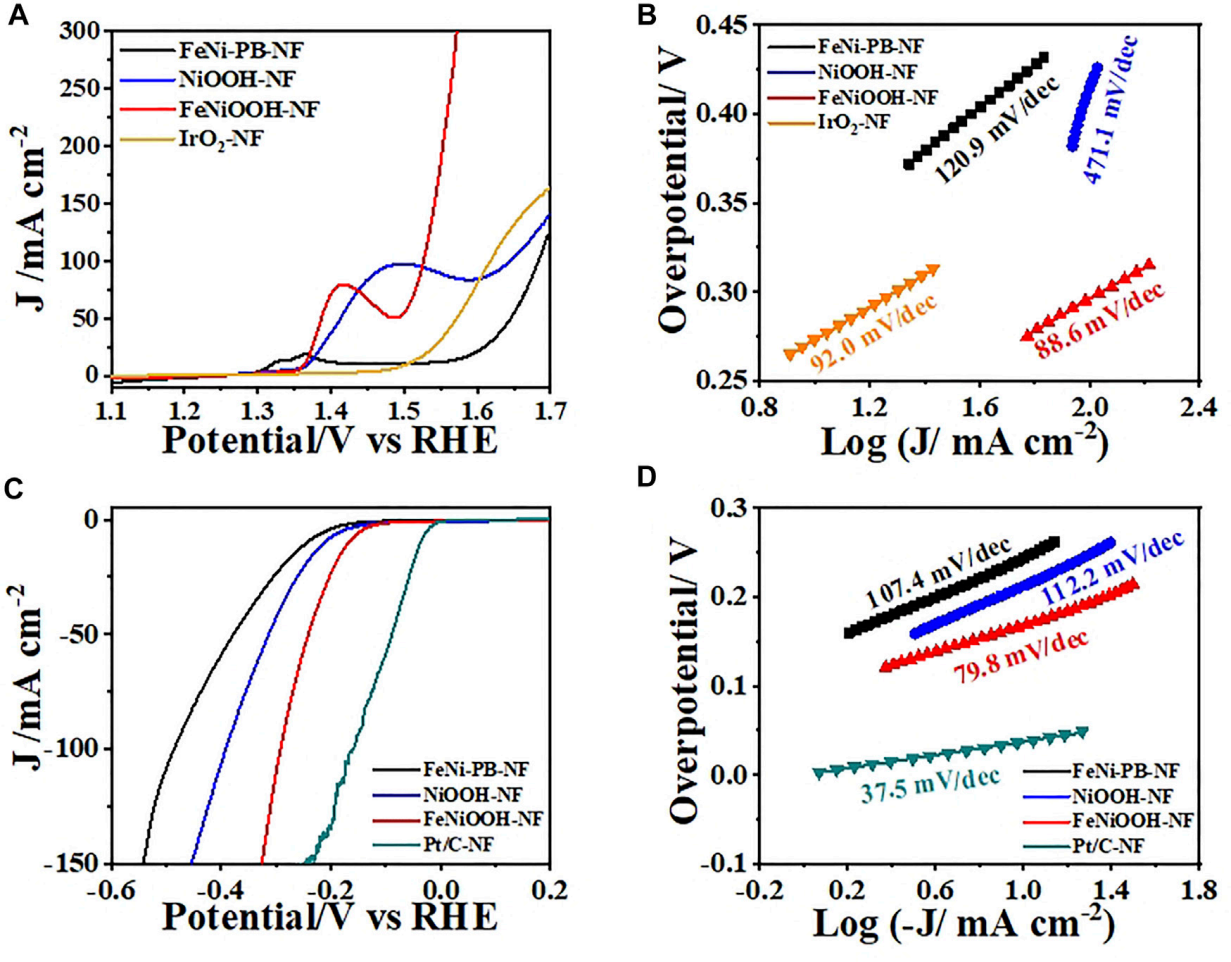
**(A)** LSVs and **(B)** Tafel plots of FeNi-PB-NF, NiOOH-NF, FeNiOOH-NF and IrO2-NF for OER. **(C)** LSVs and **(D)** Tafel plots of FeNi-PB-NF, NiOOH-NF, FeNiOOH-NF, and IrO_2_-NF for HER.

To explore the influence of material composition and structure toward electrocatalysis performance of water splitting, we carried out linear sweep voltammetry (LSV) measurements to evaluate the OER activity of FeNi-PB-NF, NiOOH-NF, FeNiOOH-NF, and IrO_2_-NF in 1 M KOH. The tests were conducted in 1 M KOH solution at 5 mV s^−1^ after iR correction in a standard three-electrode cell. It is more meaningful to compare the overpotential at large current density for practical applications. Therefore, as shown in Figure 4A, FeNiOOH-NF acquired the current density of 50 mA cm^−2^ at 1.490 V (η = 260 mV) and 100 mA cm^−2^ at 1.526 V (η = 296 mV), respectively. The overpotential at 100 mA cm^−2^ of FeNiOOH-NF was 120 mV lower than that of NiOOH-NF (1.646 V), which showed the benefits of Fe^3+^ doping to enhance the OER as reported in many previous works (Stevens et al., 2017; Bo et al., 2020; Anantharaj et al., 2021). The value was much lower than the state-of-art IrO_2_ (386 mV) and FeNi-PB-NF (453 mV). The OER kinetics of all catalysts was further reflected by their Tafel plots deriving from corresponding polarization curves. As shown in Figure 4B, the Tafel slope of FeNiOOH-NF was 88.6 mV dec^−1^, which was much lower than that of NiOOH-NF (471.1 mV/dec), FeNi-PB-NF (120.9 mV/dec) and IrO_2_-NF (92.0 mV/dec), indicating more rapid electron transfer toward improved reaction rate. The lowest overpotential and the smallest Tafel slope of FeNiOOH-NF demonstrated that the FeNiOOH-NF possessed the most efficient electrocatalytic performance for OER in this work.

The electrocatalytic HER with FeNiOOH-NF electrode was also investigated by LSV in 1.0 m KOH solution using a typical three-electrode cell. As shown in Figure 4C, although the overpotential of FeNiOOH-NF at −50 mA/cm^2^ was 240 mV, which was higher than that of Pt/C-NF (90 mV), its value was much lower than that of NiOOH-NF (315 mV) and FeNi-PB-NF (380 mV). However, the overpotential of FeNiOOH-NF became more close to that of Pt/C-NF as the current density increased, which may due to the 3D interconnected nanosheets with porous architecture FeNiOOH-NF for fast mass transport. The reduced overpotential of FeNiOOH-NF compared to NiOOH-NF indicated that the incorporation of Fe^3+^ into NiOOH also improves the HER activity. Meanwhile, the overpotential of FeNiOOH-NF was also smaller than many reported FeNiOOH materials (Liu et al., 2017; Dong et al., 2021). The reduced overpotential of FeNiOOH-NF compared to NiOOH-NF indicated that the incorporation of Fe^3+^ into NiOOH also improves the HER activity. The electrocatalytic activity for the HER could also be evaluated by comparing the Tafel slope, as shown in Figure 4D. The FeNiOOH-NF delivered a low Tafel slope of 79.8 mV dec^−1^, superior to that of NiOOH-NF (112.2 mV dec^−1^) and FeNi-PB-NF (107.4 mV dec^−1^), illustrating a rapid HER rate. The lower overpotential and the smaller Tafel slope of FeNiOOH-NF for HER showed its promising potential for electrochemical water splitting.

**FIGURE 5:**
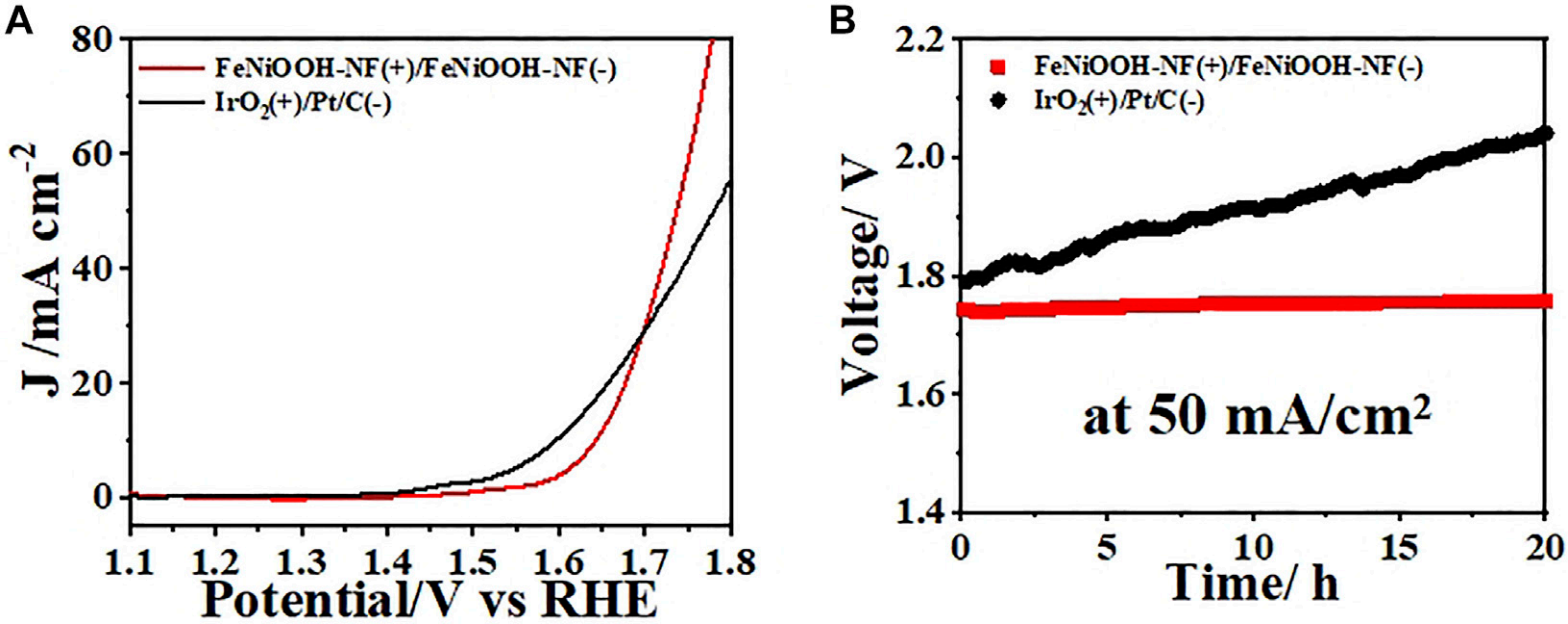
**(A)** Steady-state polarization curves for overall water splitting of bifunctional FeNiOOH-NF electrodes and IrO_2_-NF and Pt/C-NF benchmark. **(B)** Chronoamperometry tests of FeNiOOH NF eletrodes and IrO2-NF and Pt/C-NF benchmark at a current density of 50 mA cm^−2^ for 20 h.

Encouraged by the excellent OER and HER performances of FeNiOOH-NF, we assembled a two-electrode electrolyzer using FeNiOOH-NF as both cathode and anode to evaluate its potential application for electrochemical water splitting. IrO_2_ and Pt/C were loaded on NF as anode for OER and cathode for HER, respectively. The LSV curve of the electrolyzer showed that only small voltage of 1.74 V was required to obtain a current density of 50 mA cm^−2^ (Figure 5A), which was comparable to most reported FeNi-based materials (Supplementary Table S1). Although the voltage requirement of FeNiOOH-NF was larger than that of IrO_2_-NF and Pt/C-NF benchmark at low current densities (e.g., 10 mA cm^−2^), the voltage requirement to drive 50 mA cm^−2^ of FeNiOOH-NF was lower than that of IrO_2_-NF and Pt/C-NF (1.78 V), showing the benefits of 3D interconnected nanosheets with porous architecture for fast mass transport. As shown in the Supplementary Figure S3, during water splitting at 50 mA cm^−2^, hydrogen and oxygen bubbles were accumulated on the surface of NF and could be clearly observed from the optical photo. To examine the durability of FeNiOOH-NFs during electrocatalytic water splitting, a constant current density of 50 mA/cm^2^ was applied to the electrolyzer for 20 h (Figure 5B). The voltage of this electrolyzer remained extremely stable with only 0.02 V voltage loss during the stability test. However, the voltage of IrO_2_-NF and Pt/C-NF benchmark increased from 1.78 V to 2.05 V during 20 h electrolysis. Above results demonstrated the feasibility of using the as-synthesized FeNiOOH-NF as bifunctional electrodes for electrocatalytic water splitting system.

## Conclusion

In summary, we reported a unique and sustainable chemical oxidation approach for obtaining FeNiOOH nanosheets on NF as bifunctional free-standing electrodes for electrocatalytic water splitting. In the processes, FeNi-PB nanoparticles could *in situ* grow on NF and doping with Fe^3+^ ion by ion exchange. Further fast oxidation with NaClO solution (30 min) directly induced the structure reconstruction into FeNiOOH nanosheets on NF. The as-prepared self-standing FeNiOOH/NF electrode with hierarchical nanostructures exhibited excellent OER and HER performance, with low overpotentials of 260 mV and 240 mV at a current density of 50 mA cm^−2^ for OER and HER, respectively, and fast kinetic performance in 1.0 M KOH solution. Therefore, the two-electrode configuration alkaline water electrolyzer with bifunctional FeNiOOH/NF electrode as both anode and cathode delivers 50 mA cm^−2^ under a cell voltage of 1.74 V with remarkable stability, which outperform the IrO_2_-Pt/C-based electrolyzer. This work not only provides a cost-efficient surface engineering method to obtain binder-free, self-made bifunctional electrocatalyst on commercial NF, but also provides a novel strategy for their potentially scalable preparation at low cost.

## Data Availability

The original contributions presented in the study are included in the article/Supplementary Material, further inquiries can be directed to the corresponding authors.
