# Supplementary material for: Interfacial Assemble of Prussian Blue Analog to Access Hierarchical FeNi (oxy)-Hydroxide Nanosheets for Electrocatalytic Water Splitting
Source: Front Chem. 2022 Apr 27;10:895168. doi: 10.3389/fchem.2022.895168 (PMC9091355; doi:10.3389/fchem.2022.895168)
Supplement: Supplementary file 1 [file DataSheet1.zip › Supplementary Figures/Supplementary Table/Supplementary Table.docx]

**Table S1**. Comparison of overall water splitting performance of FeNiOOH materials in recent reports.

| Catalyst | Synthesis method | Potential at 50 mA cm^−2^ (V) | Substrate | Reference |
| --- | --- | --- | --- | --- |
| FeNiOOH-NF | chemical oxidation | 1.74 | Ni foam | This work |
| NiFeOOH | electrospraying | ~1.76 | Ni foam | J. Mater. Chem. A, 2021, 9, 20058 |
| S-NiFeOOH | hydrothermal | ~1.68 | Ni foam | J. Ener. Chem, 2022, 64, 364 |
| NiFeOOH/NF | hydrothermal | ~1.75 | Ni foam | I. J. H. E, 2017, 42, 5560 |
| NiFe-OH-PO_4_/NF | electrodeposition | ~1.79 | Ni foam | ACS Appl. Mater. Int, 2017, 9, 41, 35837 |
| NiFe LDH@Ni NTAs | electrochemical dealloying | ~1.76 | Ni foam | J. Power. Sources., 2020, 227434, |
| NiFe-LDH/ Ni(OH)_2_ | electrodeposition | ~1.74 | Ni foam | Chem. Engin. Jour., 2021, 419, 129608 |
