# Supplementary figures and images for: Interfacial Assemble of Prussian Blue Analog to Access Hierarchical FeNi (oxy)-Hydroxide Nanosheets for Electrocatalytic Water Splitting
Source: Front Chem. 2022 Apr 27;10:895168. doi: 10.3389/fchem.2022.895168 (PMC9091355; doi:10.3389/fchem.2022.895168)

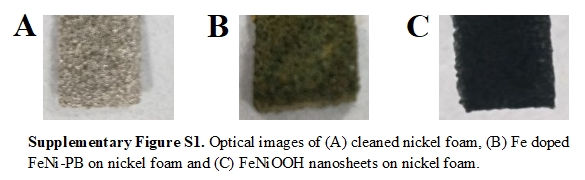

Supplement: Supplementary file 1 [file DataSheet1.zip › Supplementary Figures/Supplementary FigureS1.jpg]

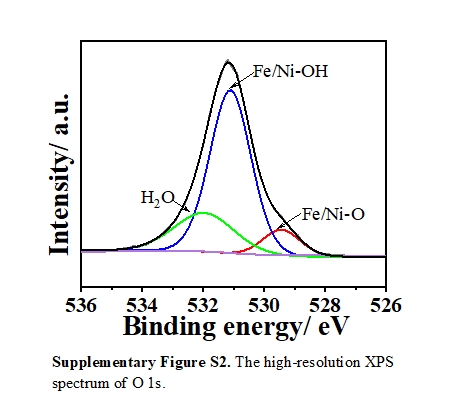

Supplement: Supplementary file 1 [file DataSheet1.zip › Supplementary Figures/Supplementary FigureS2.jpg]

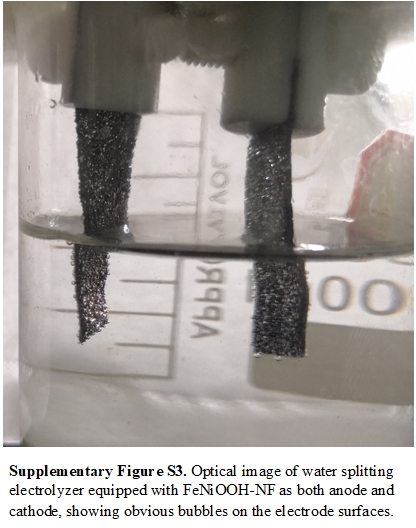

Supplement: Supplementary file 1 [file DataSheet1.zip › Supplementary Figures/Supplementary FigureS3.jpg]
